# 2773. Brazilian Bacteriophage Activity Against Multidrug-Resistant (MDR) Klebsiella pneumoniae Isolated from Patients with Severe Nosocomial Infection

**DOI:** 10.1093/ofid/ofad500.2384

**Published:** 2023-11-27

**Authors:** Nicole M Hitchcock, Danielle Nunes, Josiane Dantas, Leticia Rodrigues, Milena Soares, Roberto Badaro, David Pride

**Affiliations:** University of Missouri, Carthage, Missouri; University Center, Senai Cimatec, Salvador, Bahia, Brazil; University Center, Senai Cimatec, Salvador, Bahia, Brazil; University Center, Senai Cimatec, Salvador, Bahia, Brazil; University Center, Senai Cimatec, Salvador, Bahia, Brazil; University Center, Senai Cimatec, Salvador, Bahia, Brazil; Department of Infectious Diseases and Global Public Health, University of California San Diego, La Jolla, CA, San Diego, California

## Abstract

**Background:**

Phage therapy has re-emerged as a promising strategy for combatting antimicrobial resistance. Phages are ubiquitous viruses that infect and destroy bacteria and are easily isolated from environmental samples. An impressive reservoir of potentially efficacious bacteriophages has yet to be discovered, and the isolation of novel phages from diverse environments is of global importance.

**Methods:**

A phage research lab was implemented in Salvador Bahia, Brazil, in collaboration with the Center for Innovative Phage Applications and Therapeutics (IPATH) located in San Diego, California. The goal of this project was to discover and isolate phages from water systems throughout the area. Samples were collected from sources including standing water surrounding residences in impoverished communities (*favelas*), sewage, and the intersection of seawater and wastewater along the coast. Protocols were followed for phage enrichment, spot assay, isolation, and purification utilizing the double-layer agar method. Phages isolated against laboratory strain *K. pneumoniae* (ATCC 10031) were then spot-tested against 30 clinical, multidrug-resistant strains of *K. pneumoniae* which were collected from a local hospital.

**Results:**

A total of 28 phages were isolated from environmental samples with efficacy against the host strain, *K. pneumoniae.* All 28 phages were then tested against the 30 clinical strains of MDR *K. pneumoniae*, among which nine had at least intermediate activity against five or more MDR *K. pneumoniae* strains based on qualitative spot assay results. Two of the clinical *K. pneumoniae* samples were ESBL+ and showed at least intermediate sensitivity to three and four phages, respectively. Of the 30 clinical bacterial samples, six were completely resistant to all 28 phages. 25 of the tested phages revealed activity against at least one clinical *K. pneumoniae* strain.

Spot assay demonstrating phage activity against host strain K. pneumoniae

**Figure:**
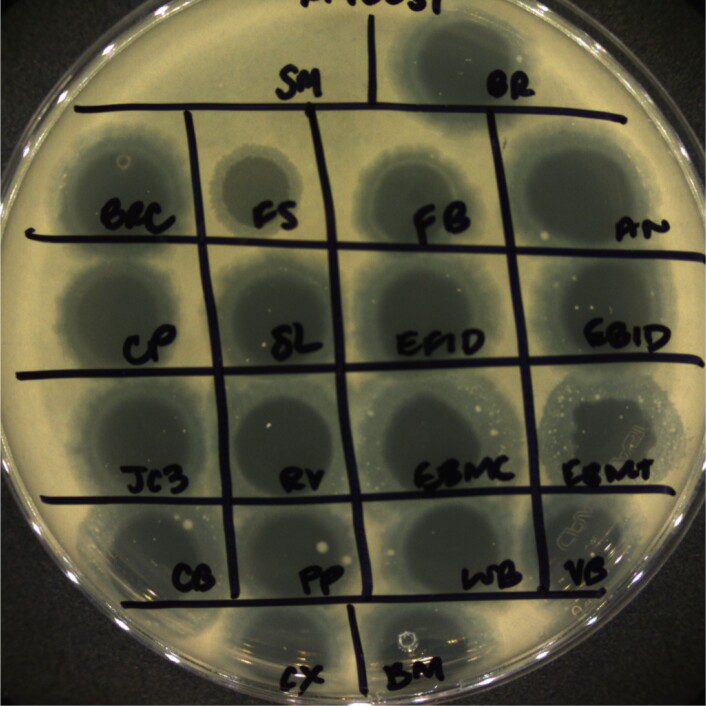

19 of the 28 phages are shown spotted against a double-layer agar plate poured with host strain K. pneumoniae (ATCC 10031). All phage spots reveal disruption and clearing of the bacterial lawn, indicating bacterial sensitivity to each respective phage. Control spot, "SM" included only SM buffer and no phage for comparison.

Brazilian Bacteriophage Activity Against Clinical K. pneumoniae

**Figure:**
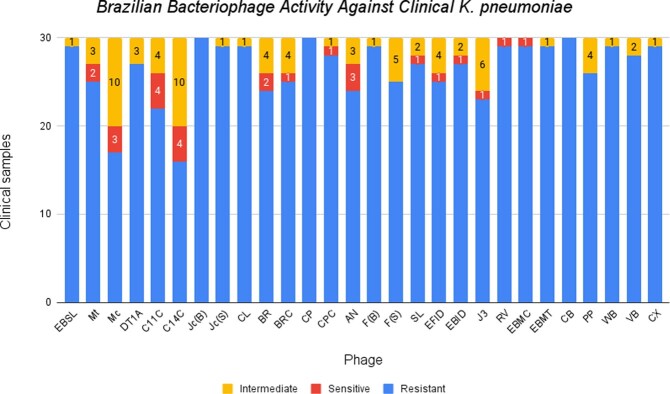

Results of the spot assays performed with all clinical strain K. pneumoniae were qualitatively described as resistant, sensitive, or intermediate. The majority of tested bacteriophages (25) revealed some disruption of the bacterial lawn. Three bacteriophages (Jc(B), CP, and CB) did not demonstrate activity against any clinical bacterial samples. Phage C14C demonstrated activity against the greatest number of clinical bacteria (14 total intermediate/sensitive).

Individual Sensitivity of Clinical Klebsiella pneumoniae (CKP) Samples to Brazilian Phages

**Figure:**
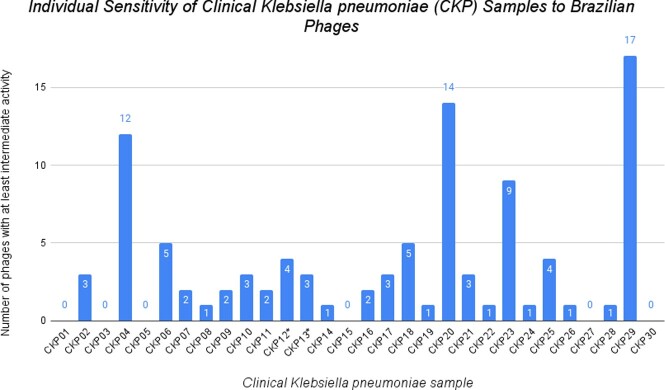

* Refers to ESBL-positive clinical Klebsiella pneumoniae. Six of the thirty CKP samples were resistant to all 28 bacteriophages. CKP29 was the most sensitive to the tested phages, with either intermediate or complete sensitivity to 17 phages. The majority of CKPs (20) were at least intermediately sensitive to between one and five phages.

**Conclusion:**

There is potential to isolate phages from highly contaminated wastewaters in Brazil which have the capability to infect and kill distinct strains of *K. pneumoniae* in the laboratory setting. Continued international collaboration will be imperative in the global fight against antimicrobial resistance.

**Disclosures:**

**All Authors**: No reported disclosures

